# Protective Effects of Different Kinds of Filtered Water on Hypertensive Mouse by Suppressing Oxidative Stress and Inflammation

**DOI:** 10.1155/2018/2917387

**Published:** 2018-12-02

**Authors:** Qian Sun, Fan Xin, Xinan Wen, Chan Lu, Ronghe Chen, Guohong Ruan

**Affiliations:** ^1^Department of Health Inspection and Quarantine, School of Public Health, Fujian Medical University, Fuzhou 350122, China; ^2^Department of Nutrition, The Second Affiliated Hospital of Wenzhou Medical University, Wenzhou 325000, China; ^3^State Key Laboratory of Molecular Vaccinology and Molecular Diagnosis & Center for Molecular Imaging and Translational Medicine, School of Public Health, Xiamen University, Xiamen 361102, China

## Abstract

Oxidative stress and inflammation play an important role in hypertensive animals and patients. Hydrogen plays a role of antioxidation and anti-inflammation. Calcium and magnesium play an important role in reducing hypertension and antioxidant. Filtered water contains abundant hydrogen and a large number of other essential elements of the human body. We investigated the protective effects of filtered water on hypertensive mice. To establish hypertension model, ICR mice were administered with N′-nitro-L-arginine methyl ester (L-NAME) hydrochloride 64 mg/kg per day for 1 month. The hypertensive mice were, respectively, administered with pure water, tap water, and filtered water for 2 months. Lipid peroxidation, antioxidant enzymatic activity, endothelin-1 (ET-1), angiotensin II (Ang II), and proinflammatory cytokines (TNF-*α*, IL-6, and IL-1*β*) were assessed. Expressions of phosphorylated NF-*κ*B P65 in the kidney were analyzed by western blot. qRT-PCR analysis was adopted to determine the expression levels of the proinflammatory cytokines and NF-*κ*B P65. The results demonstrated that filtered water can reduce the blood pressure. Filtered water treatment restored the activity of antioxidant enzymes, downregulated ET-1, and Ang II in the serum of mice. Filtered water treatment suppressed proinflammatory cytokines and decreased the mRNA expression of TNF-*α*, IL-6, IL-1*β*, and NF-*κ*B P65. Consumption of filtered water inhibited the expression of NF-*κ*B P65. This suggests that filtered water can reduce the blood pressure. The protection mechanisms include downregulating oxidative stress and inhibiting inflammation, which is partly due to the inhibition of the NF-*κ*B signaling pathway.

## 1. Introduction

Hypertension is one of the major causes of morbidity and mortality, affects a considerable proportion of the population, and has become a significant public health problem [1]. Currently, antihypertensive treatments are mostly drugs. Previous studies mainly focused on the use of vasodilators [2–4], including the inhalation of nitric oxide, and the use of cyclic guanosine monophosphate-generating agents and endothelin receptor antagonists. This medication is expensive and has a lot of side effects. Therefore, it is imperative to look for the additional novel therapies.

The pathogenesis of hypertension has not yet been defined. Hypertension may share some of the following pathological or functional changes [5], including vascular remodeling, endothelial dysfunction/increased vasoconstriction, oxidative stress, and inflammation. Studies [6, 7] have demonstrated that reactive oxygen species (ROS) plays an important role in the occurrence of hypertension. Oxidative stress produced by overproduction of ROS or inefficient antioxidant defenses appears to be involved in the development and progression of hypertension [8]. Furthermore, the mechanisms of inflammation in hypertension include upregulation of cytokines.

Some reports have displayed that calcium and magnesium play an important role in reducing hypertension and antioxidant [9–12]. However, there were few reports of the effects of filtered water on blood pressure. Compared with other kinds of water, filtered water (FW) has properties of essential elements than other water, negative oxidation-reduction potential (ORP) level and alkaline pH, which are produced by filtering the tap water through certain water purifier. The water purifier contains 5 filter elements: polypropylene fusible spray filter element, ceramic composite filter, carbon filter, composite filter, and active carbon filter. Composite filter contains medicinal stone “maifanshi.” FW is repeatedly filtered by maifanshi. Its mineral and microelement content far exceeds that of any ordinary water. Different from filtered water, pure water (PW) is produced through 4 filtration processes of tap water: sedimentation, precarbon, reverse osmosis membrane, and postcarbon filtration, and it contains the fewest elements. Previous studies on electrolyzed reduced water and alkaline reduced water, which have the same characteristics of filtered water, showed beneficial effects such as prevention of liver inflammation [13].

Therefore, we hypothesize that the filtered water might prevent the progression of hypertension by antioxidant and anti-inflammatory. In this study, we investigated the effect of different kinds of drinking water on the hypertension mice and further explored its possible mechanism.

## 2. Materials and Methods

### 2.1. Animals

A total of 70 adult male ICR mice were purchased from Fujian Medical University. After a one-week acclimatization, the mice were randomly divided into 2 groups: the control group (Con) (*n* = 16) and the model group (*n* = 54). The mice in the model group were fed with N′-nitro-L-arginine methyl ester (L-NAME) hydrochloride 64 mg/kg per day [14], and the control group was treated with the same dose of pure water. 1 month later, the mice that have been successfully modeled (SBP > 95% CI of the control group) (*n* = 45), randomly divided into 3 groups: the pure water (PW) group (*n* = 15), the tap water (TW) group (*n* = 15), and the filtered water (FW) group (*n* = 15), were given the corresponding drinking water for 2 months. The control group received pure water.

All the animals were housed under constant conditions of temperature (23 ± 2°C) and illumination (light 08:00-20:00 h; darkness 20:00-08:00 h). Animals had free access to food and water. The water and food intake of the mice were recorded once every two days. The animal protocols were performed in accordance with the guidelines with approval of the Animal Experiment Ethics Committee at Fujian Medical University.

### 2.2. Blood Pressure Measurement

Blood pressure (BP) and heart rate (HR) were assessed in conscious mice using a standard tail-cuff technique. The mice were mildly warmed up for 30 min prior to BP assessment. Only BP measurements from resting animals were considered. Three readings were recorded and averaged to obtain the mean values.

### 2.3. Water Quality Analysis

The water quality parameters were measured for three kinds of drinking water, and the parameters included total dissolved solids (TDS), pH, oxidation reduction potential (ORP), electric conductivity (EC), dissolved hydrogen, calcium (Ca), and magnesium (Mg). The TDS, pH, ORP, and EC were measured by multiparameter water-detecting instrument. The dissolved hydrogen was measured by the experimental test pen. The calcium and magnesium were measured by flame atomic absorption spectrophotometer, according to the national standard detection for drinking water (China) (GB/T 5750-2006).

### 2.4. Serum Biochemical and Immunological Analysis

2 months later, after12 h of fasting, mice were anaesthetized with sodium pentobarbital (100 mg/kg) and sacrificed. Blood was collected. After centrifugation at 3500 rpm for 10 min at 4°C, the serum was collected and serum total superoxide dismutase (T-SOD), malondialdehyde (MDA), glutathione peroxidase (GSH-Px), glutathione S-transferase (GSH-ST), nitric oxide (NO), and nitric oxide synthase (NOS) (Jiancheng Tech, Nanjing, China) were analyzed according to the manufacturer's instructions. Serum interleukin-6 (IL-6), interleukin-1*β* (IL-1*β*), tumor necrosis factor-*α* (TNF-*α*), endothelin-1 (ET-1), and angiotensin II (Ang II) were detected by enzyme-linked immunosorbent assay (ELISA) kits (Tongwei, Shanghai, China).

### 2.5. Measurement of Lipid Peroxidation and Antioxidant Enzymatic Activity in the Kidney

The kidney was weighed (100 mg) and homogenized in 900 *μ*L cold PBS (pH 7.4) by homogenizer. After centrifugation at 3000 rpm for 10 min at 4°C, the supernatant was collected. Tissue T-SOD, MDA, GSH-Px, and GSH-ST were measured by commercial assay kits (Jiancheng Tech, Nanjing, China) according to the manufacturer's protocols. The absorbance values were measured by a microplate reader (Molecular Devices, USA).

### 2.6. Quantitative Real-Time PCR (qRT-PCR) Analysis of the Kidney

The expression levels of TNF-*α*, IL-1*β*, IL-6, and NF-*κ*B P65 were analyzed via qRT-PCR. Briefly, total RNA was isolated from renal tissues with TRIzol reagent (Thermo Fisher Scientific, USA) according to the manufacturer's protocol and reversely transcribed into cDNA using reverse transcription kits (Takara Bio, Japan). Real-time PCR analysis was performed with a QuantiTect™ SYBR Green PCR (Takara Bio, Japan) according to the manufacturer's instructions. The primers (Table 1) were synthesized by Shanghai Biological Engineering (Shanghai, China). Relative quantification of the target gene expression levels was conducted using the 2^−∆∆Ct^ method.

### 2.7. Western Blot Analysis

A BCA kit (Beyotime, Shanghai, China) was utilized in order to quantify the amount of protein. Equal amount of protein preparations was run on SDS-polyacrylamide gels, electrotransferred to polyvinylidene difluoride membranes, and blotted with anti-P65 (Cell Signaling Technology, Boston, USA) overnight at 4°C using slow rocking. Then, they were blotted with HRP-conjugated secondary antibody (Servicebio, China). The antibody-antigen complexes were visualized by using the electrochemiluminescence (ECL) kit (Servicebio, China). The protein bands were detected with ChemiScope 6300 (Clinx Science Instruments Co. Ltd, China). The bands were analyzed with the AlphaEaseFC software and compared with *β*-actin.

### 2.8. Statistical Analysis

The SPSS 19.0 and GraphPad Prism 6 were used for data analysis. Data were expressed as mean ± standard deviation (SD). Mean values for each group were analyzed using a one-way ANOVA. Intergroup comparative analysis was facilitated by LSD and tested with *P* < 0.05 being regarded as statistically significant.

## 3. Results

### 3.1. Water Quality Parameters

Filtered water has properties of high dissolved hydrogen, negative ORP level, and alkaline pH. The TDS, EC, Ca, and Mg in filtered water were higher than that in tap water. EC, TDS, Ca, and Mg in pure water were lower than the detection limits (Table 2).

### 3.2. The FW Treatment Reduced the Blood Pressure in Hypertensive Mice

Compared to the Con, SBP, MBP, and DBP in mice that were given L-NAME were significantly increased, indicating that the mice have developed severe hypertension. Treatment of mice with the FW for 2 months, the SBP, MBP, and DBP were significantly decreased compared to the PW and TW, whereas the SBP, MBP, and DBP of the mice in the PW and TW were significantly higher than that in the Con. There were no significant differences in HR in four groups (Table 3).

### 3.3. The Changes of the Enzymes in the Serum of Mice

**Figure 1:**
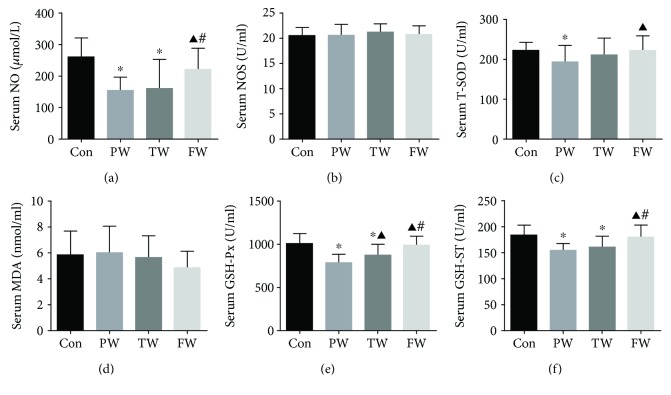
The effect of drinking water on serum enzymes. The mice were sacrificed, blood was collected, and serum was separated. Serum NO (a), NOS (b), T-SOD (c), MDA (d), GSH-Px (e), and GSH-ST (f) were detected. Data were presented as mean ± SD; ^∗^*P* < 0.05 versus Con, ^▲^*P* < 0.05 versus PW, and ^#^*P* < 0.05 versus TW.

The content of NO and GSH-ST in the PW and TW was significantly lower than that in the Con, and the concentrations in the FW increased significantly as compared with that in the PW and TW (Figures 1(a) and 1(f)). The serum T-SOD in the PW decreases significantly compared with that in the Con while the FW treatment resulted in marked increase compared to the PW treatment. Compared to the Con, GSH-Px decreased significantly in the PW and TW. It was noted that the FW significantly increased the GSH-PX activity compared with the PW and TW (Figure 1(e)). There were no significant differences in serum NOS and MDA in each group (Figures 1(b) and 1(d)). Consumption of the FW and TW restored the activities of antioxidant enzymes in hypertensive mouse, at least in part (Figure 1).

### 3.4. The Changes of the Proinflammatory Cytokines in the Serum of Mice

**Figure 2:**
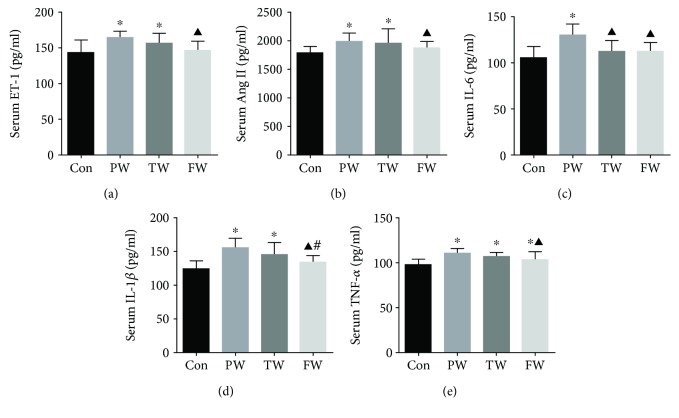
The effect of drinking water on serum inflammatory factor. The mice were sacrificed, blood was collected, and serum was separated. Serum ET-1 (a), Ang II (b), IL-6 (c), IL-1*β* (d), and TNF-*α* (e) were detected. Data were presented as mean ± SD; ^∗^*P* < 0.05 versus Con, ^▲^*P* < 0.05 versus PW, and ^#^*P* < 0.05 versus TW.

ELISA detection showed that the levels of ET-1, Ang II, IL-1*β*, and TNF-*α* in serum were evident with marked increase in the PW and TW compared to the Con, and significant reductions were noted in the FW compared with the PW (Figures 2(a) and 2(b) and 2(d) and 2(e)). Compared with the Con, IL-6 was higher in the PW, which was reversed after treatment with the TW and FW (Figure 2(c)).

### 3.5. The Changes of the Antioxidative Enzymes in the Kidney

**Figure 3:**
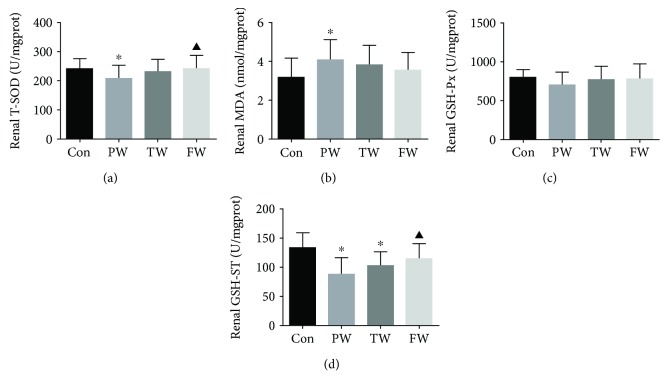
The effect of drinking water on enzyme in the kidneys. The mice were sacrificed, and the kidneys were separated. Renal T-SOD (a), MDA (b), GSH-Px (c), and GSH-ST (d) were detected. Data were presented as mean ± SD; ^∗^*P* < 0.05 versus Con and ^▲^*P* < 0.05 versus PW.

The content of T-SOD in the PW was lower than that in the Con. Compared to the PW, T-SOD of the mice in the FW increased significantly (Figure 3(a)). MDA was markedly higher in the PW than in the Con, but there was no evident difference in the TW and FW when compared to the PW and Con (Figure 3(b)). Additionally, GSH-ST was significantly lower in the PW and TW than in the Con, whereas GSH-ST was restored by consumption of the FW (Figure 3(d)). There were no significant differences in GSH-Px in the PW, TW, FW, and Con (Figure 3(c)).

### 3.6. The Changes of the mRNA Expressions in the Kidney

**Figure 4:**
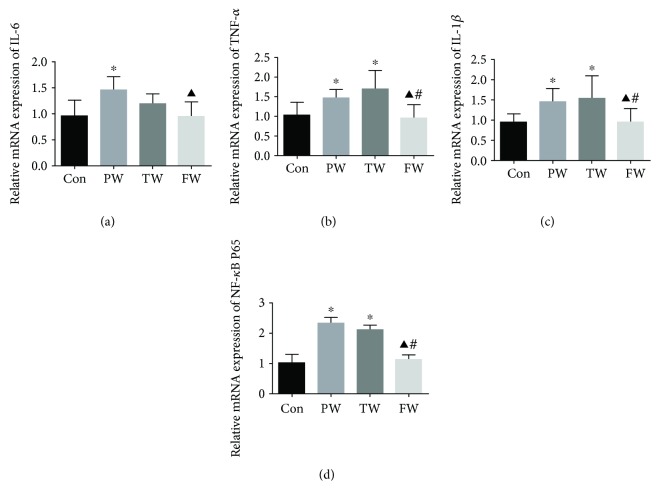
The effect of drinking water on the expression of inflammatory factors in the kidney. IL-6 (a), TNF-*α* (b), IL-1*β* (c), and NF-*κ*B P65 (d) mRNA expression was qualified by qRT-PCR. Data were presented as mean ± SD; ^∗^*P* < 0.05 versus Con, ^▲^*P* < 0.05 versus PW, and ^#^*P* < 0.05 versus TW.

IL-6, TNF-*α*, IL-1*β*, and NF-*κ*B P65 are abundantly expressed in the kidney. The PW and TW exhibited higher expression of TNF-*α*, IL-1*β*, and NF-*κ*B P65 compared to the Con. Treatment with the FW, they were restored (Figures 4(b)–4(d)). The mRNA expression levels of IL-6 in the PW were higher than those in the Con (Figure 4(a)). The FW reduced the mRNA level of IL-6. There was no significant difference in IL-6, TNF-*α*, IL-1*β*, and NF-*κ*B P65 between the PW and TW.

### 3.7. FW Treatment Downregulated the Protein Expression Levels of Phosphorylated NF-*κ*B P65

**Figure 5:**
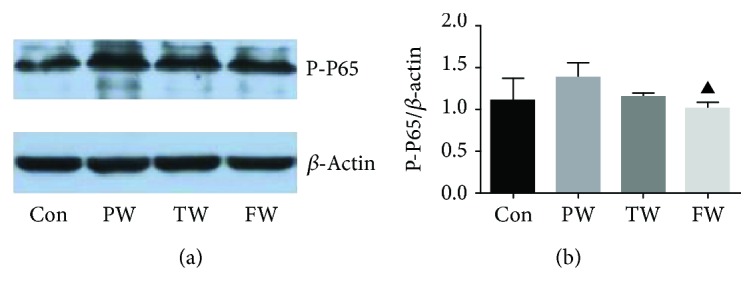
The effect of drinking water on the expression of protein in the kidney. (a) Representative western blot and (b) quantitative analyses revealing the effects of different kinds of water on the expression of phosphorylated NF-*κ*B P65 in the kidney. *β*-Actin was used to ensure that an equal amount of protein was loaded in each lane. Data were presented as mean ± SD; ^▲^*P* < 0.05 versus PW.

As a subunit of the NF-*κ*B dimer, P65 has typically been chosen as an index of NF-*κ*B activation. The PW exhibited higher expression of phosphorylated NF-*κ*B P65 in the kidney compared to the Con, which was demonstrated by western blotting analysis. Consumption of the FW decreased the expression level of phosphorylated NF-*κ*B P65 in the kidney (Figure 5).

## 4. Discussion

Increasing evidence has demonstrated that oxidative stress plays an important role in the occurrence and development of hypertension [18]. Malondialdehyde is the ultimate product of unsaturated lipid peroxidation. MDA is often used as a marker of oxidative damage [19, 20]. The measurement of MDA in the blood may provide information on an excessive generation of free radical-induced membrane injury. SOD, GSH-Px, and GSH-ST are important antioxidant enzymes in the regulation of oxidative damage. In this study, it is found that the level of MDA was increased, and the activity of T-SOD, GSH-Px, and GSH-ST was decreased in mice in the PW compared to the control group. It suggests that oxidative damage occurred in hypertensive mice. In contrast, the FW treatment significantly increased T-SOD and GSH-ST activity, which is consistent with its antioxidative effect. The level of MDA decreased in serum and in the kidney. In this study, consumption of the FW not only restored the activity of antioxidant enzymes but also reduced the lipid peroxidation products.

This study finds that apart from antioxidative property, the FW also exerted an anti-inflammatory property. Inflammation contributes to the progression of hypertension, whereas ROS can induce inflammatory cytokine activities via the assistance of the NF-*κ*B pathway [21]. Excess ROS overwhelms antioxidant defenses, leading to oxidative stress. ROS mediates several biochemical and molecular pathways that can exacerbate oxidative stress [22], such as activating the transcription factor nuclear factor kappa B (NF-*κ*B) which increases the transcription of inflammatory cytokines and chemokines promoting inflammation. In this study, serum IL-6, IL-1*β*, and TNF-*α* levels were upregulated significantly in the PW and TW, while the serum IL-6, IL-1*β*, and TNF-*α* levels were downregulated significantly by treatment with the FW. The mRNA expression of the proinflammatory cytokines (IL-6, IL-1*β*, and TNF-*α*) were significantly decreased in the kidney in the FW. These results suggest that the effects of the FW on hypertension might be mediated by depression of IL-6, IL-1*β*, and TNF-*α*, and the FW has an anti-inflammatory activity. Collectively, these data were consistent with the hypothesis that the FW inhibits the production of inflammatory factors via the NF-*κ*B signaling pathway. Here, it is found that treatment with the FW suppressed proinflammatory cytokines including IL-6, TNF-*α*, and IL-1*β*. The effect of anti-inflammatory of the hydrogen might be inferior to its antioxidative effect. Excess ROS activates the redox-sensitive transcription factor NF-*κ*B, resulting in the enhancement of its expression and activity [21, 23]. Increased expression and activity of NF-*κ*B induce gene transcription of proinflammatory cytokines, such as IL-6 and IL-1*β*, to increase their production [21, 24]. In this study, renal NF-*κ*B activation was inhibited by treatment with the FW, which might interpret the anti-inflammatory property of the FW.

Some reports display that calcium and magnesium play an important role in reducing hypertension and antioxidation [25]. Calcium can enhance the ability of antioxidant and anti-inflammatory. High-calcium diet can inhibit the hypertension and increase the levels of NO and NOS in hypertensive rats absent of NO [26]. Magnesium can significantly reduce the content of MDA, inhibit the formation of free radicals, and promote the scavenging effect of free radicals [27]. The study of tissue [28], plasma lipoprotein [12], and the liver of the mice [29] shows that the absence of magnesium can increase the degree of lipid peroxidation in tissues, whereas the increased magnesium results in higher content of GSH in red blood cells. Lv [30] study of the effects of Mg^2+^ on the expression of redox-related genes c-fos, c-jun, P53, and Ref-1 from the genetic level by immunohistochemistry technique displayed that magnesium inhibits the expression of gene that is related to the redox. In our study, we found that the contents of calcium and magnesium from high to low were observed, respectively, in filtered water, tap water, and pure water. We speculate that the decrease of blood pressure in mice may be related to the content of calcium and magnesium in water.

Hydrogen gas (H_2_) has been applied in medical application to prevent decompression sickness [31]. Some reports displayed that the water which dissolved large amounts of hydrogen had the ability to protect DNA from oxidative damage [32]. Hydrogen water played a protection effect on pulmonary hypertension by antioxidation [33]. Moreover, it has been reported that hydrogen-rich saline has an anti-inflammatory effect [34]. Filtered water has properties of high dissolved hydrogen, negative oxidation-reduction potential (ORP) level, and alkaline pH. Therefore, we hypothesize that the descending of pressure has to do with the hydrogen in the FW.

This study shows that FW treatment ameliorates the blood pressure in mice, which may be associated with the effects of its anti-inflammatory and antioxidant. Our findings suggest that the FW may be beneficial to the treatment of hypertension. The beneficial effect of this treatment is a result of its ability to relieve oxidative stress and inflammation and may be mediated by the complex modulation of the NF-*κ*B signaling pathways. Consumption of the FW reduces blood pressure, indicating that it is a promising strategy for antihypertensive therapy.

## 5. Conclusion

This study suggests that filtered water can reduce blood pressure. The mechanisms of the protection include downregulating oxidative stress and inhibiting inflammatory, and it is partly due to the inhibition of the NF-*κ*B signaling pathway.

## Figures and Tables

**Table 1:** Sequences of the primers used for PCR [15, 16].

Genes	Forward primer	Reverse primer
IL-6	TAGTCCTTCCTACCCCAATTTCC	TTGGTCCTTAGCCACTCCTTC
TNF-*α*	CCCTCACACTCAGATCATCTTCT	GCTACGACGTGGGCTACAG
IL-1*β*	GCAACTGTTCCTGAACTCAACT	ATCTTTTGGGGTCCGTCAACT
NF-*κ*B P65	ATGATCGCCACCGGATTGA	GAGTTTCGGGTAGGCACAGCA
GAPDH	AGGTCGGTGTGAACGGATTTG	TGTAGACCATGTAGTTGAGGTCA

**Table 2:** The parameters of three kinds of drinking water [17].

Group	pH	H_2_ (mg/L)	ORP (mv)	EC (ms/cm)	TDS (mg/L)	Ca (mg/L)	Mg (mg/L)
PW	6.10 ± 0.12	<0.01	216.90 ± 7.80	<0.01	<0.01	<0.05	<0.02
TW	6.57 ± 0.10	<0.01	521.70 ± 10.04	0.06 ± 0.00	48.00 ± 4.22	3.07 ± 0.05	0.53 ± 0.01
FW	7.71 ± 0.02	0.66 ± 0.03	−214.50 ± 21.12	0.13 ± 0.00	92.00 ± 4.22	6.87 ± 0.23	0.59 ± 0.01

**Table 3:** The changes of heart rate, systolic blood pressure, mean blood pressure, and diastolic blood pressure of the mice.

		HR	SBP (mmHg)	MBP (mmHg)	DBP (mmHg)
0 month later	Con	693.44 ± 48.96	108.11 ± 4.54	92.84 ± 4.31	85.05 ± 4.79
	PW	664.19 ± 47.37	120.04 ± 4.00^∗^	98.56 ± 4.77^∗^	88.83 ± 5.63^∗^
	TW	640.84 ± 73.13	120.99 ± 5.11^∗^	99.76 ± 5.29^∗^	88.91 ± 6.09^∗^
	FW	653.10 ± 60.23	119.50 ± 3.77^∗^	97.18 ± 3.59^∗^	89.24 ± 2.38^∗^
2 months later	Con	689.39 ± 60.60	106.48 ± 7.37	90.48 ± 5.18	82.35 ± 4.77
	PW	677.88 ± 44.46	115.67 ± 3.00^∗^	96.49 ± 4.72^∗^	87.41 ± 6.11^∗^
	TW	654.87 ± 62.33	114.24 ± 3.83^∗^	96.40 ± 5.03^∗^	87.33 ± 6.33^∗^
	FW	678.44 ± 50.64	104.34 ± 3.42^▲#^	88.15 ± 2.85^▲#^	80.06 ± 3.50^▲#^

^∗^ *P* < 0.05 versus Con, ^▲^*P* < 0.05 versus PW, and ^#^*P* < 0.05 versus TW.

## Data Availability

The datasets used or analyzed during the current study are available from the corresponding author on reasonable request. All data generated or analyzed during this study are included in this published article.
